# ‘Listen and learn:’ participant input in program planning for a low-income urban population at cardiovascular risk

**DOI:** 10.1186/s12889-021-10423-6

**Published:** 2021-03-15

**Authors:** Rachel S. Kirzner, Inga Robbins, Meghan Privitello, Marianne Miserandino

**Affiliations:** 1 School of Social and Behavioral Sciences, Stockton University, 101 Vera King Farris Drive, Galloway, NJ 08205 USA; 2 Atlanticare Health Services, 1401 Atlantic Ave, Atlantic City, NJ 08401 USA; 3 Sexual Assault Program, AVANZAR, 927 Main Street, Building D, Pleasantville, NJ 08232 USA; 4 Psychology, Arcadia University, 450 S Easton Rd, Glenside, PA 19038 USA

**Keywords:** Focus groups, Health disparities, Cardiovascular disease, Self-determination theory, Program planning, Peer support

## Abstract

**Background:**

Poverty increases the risk of cardiac disease, while diminishing the resources available to mitigate that risk. Available prevention programs often require resources that low-income residents of urban areas do not possess, e.g. membership fees, resources to purchase healthy foods, and safe places for physical activity. The aim of this study is to obtain participant input in order to understand the health-related goals, barriers, and strengths as part of planning a program to reduce cardiovascular risk.

**Methods:**

In a mixed methods study, we used written surveys and focus groups as part of planning an intervention specifically designed to meet the needs of lower income individuals. Based on prior research, we used Self-Determination Theory (SDT) and its core constructs of autonomy, competence, and relatedness as the theoretical framework for analysis. The study collected information on the perspectives of low-income urban residents on their risks of cardiovascular disease, their barriers to and supports for addressing health needs, and how they addressed barriers and utilized supports. Focus group transcripts were analyzed using standard qualitative methods including paired coding and development of themes from identified codes.

**Results:**

Participants had health goals that aligned with accepted approaches to reducing their cardiovascular risks, however they lacked the resources to reach those goals. We found a lack of support for the three SDT core constructs. The barriers that participants reported suggested that these basic psychological needs were often thwarted by their environments.

**Conclusions:**

Substantial disparities in both access to health-promoting resources and in support for autonomy, competence, and relatedness must be addressed in order to design an effective intervention for a low-income population at cardiac risk.

**Supplementary Information:**

The online version contains supplementary material available at 10.1186/s12889-021-10423-6.

## Background

Significant disparities persist in cardiovascular disease (CVD) mortality rates by race, ethnicity, socioeconomic status, and geographic location [1]. For instance, even in this decade, individuals who are African American have a 27% higher age-adjusted death rate from CVD than the general population [2], and persons aged 35 to 64 in the lowest quartile of socioeconomic status are twice as likely to die from myocardial infarction and coronary heart disease compared to those in the higher quartiles. These disparities mirror the significant disparities in the prevalence of seven key risk factors: smoking, physical inactivity, obesity, poor diet, hypertension, high cholesterol, and abnormal fasting glucose [3]. In addition, disparities in stressors including Adverse Childhood Experiences (ACEs) contribute to cardiovascular risk by multiple pathways [4].

Access to healthy foods and exercise, two powerful preventative factors for CVD, can be an insurmountable barrier for the most vulnerable populations. Low-income neighborhoods may lack supermarkets, a situation described as a food desert [5–7]. Gentrification of neighborhoods has resulted in food mirages: local markets that provide wide selections of fresh produce and other healthful foods, but at prices inaccessible to low-income consumers [8]. Similar to food deserts, there is a lack of access to exercise in areas of poverty, which has been termed exercise deserts [9]. In addition to poor access to healthy foods and exercise, individuals living in poverty are exposed to disproportionate environmental toxicities. Multiple studies connect the stress of living in low-income, under-resourced neighborhoods to chronic diseases [10, 11]. Elevated exposure to stressors begins in childhood, and ACEs are more common in low-income neighborhoods [12]. Increased frequency of ACEs correlates with incidence of cardiovascular disease [4].

Widely available and sustainably funded cardiac prevention programs, such as traditional cardiac rehabilitation and the Ornish Reversal Program, are very effective at reducing recurrent cardiac events [13]. However, they may only serve to increase the disparities discussed above. The lifestyle changes they promote require those same diet and exercise resources that are inaccessible to a low-income urban population. Furthermore, the providers of these programs may not be knowledgeable about the goals and lived experiences of these populations. This knowledge is essential in planning a program that will reduce disparities faced by this population. We will describe how Self-determination Theory (SDT) can further illuminate the impact of these disparities as well as many providers’ limited understanding of these populations.

Our study used focus groups and written surveys to explore the cardiovascular health-related goals, barriers, and strengths of a low-income population of urban residents. We sought this information as part of planning a peer-support program to address cardiovascular risk. Focus group research is based on valuing participant input; therefore it is not surprising that focus group studies have used SDT as a theoretical framework both in designing the studies and analyzing the results.

Because SDT identifies the three basic needs (autonomy, competence, and relatedness) that foster human motivation (likelihood of engaging in behaviors), it is inherently relevant to health behaviors [14]. In the context of health care, competence means possessing resources and knowledge, and feeling capable of taking the steps required to maintain one’s health. Autonomy refers to the choice to pursue specific health goals rather than feeling pressured to follow a doctor’s orders or by an already distrusted medical establishment [15]. Relatedness in the healthcare environment means feeling supported by health care professionals and by peers. When these three basic needs of competence, autonomy, and relatedness are met, people feel intrinsic motivation, become engaged in their own health care, and as a result have positive health outcomes [16].

This theory posits that motivation is powerfully influenced by environmental factors that support or undermine SDT needs, rather than being inherent in individuals. For example, a person’s home life, neighborhood resources, and access to health care all have the potential to support or block a person from feeling motivated to take the actions which will lead to positive health outcomes. Therefore, SDT may be especially relevant to populations with scarce resources. When resources are scarce, people are less able to get their needs of competence, autonomy, and relatedness met [17]. Providers with a limited understanding of the preferences and resources of an underserved diverse population may further undermine these three preconditions for motivation. This theory potentially provides compelling insight into why adherence to provider health behavior recommendations is thwarted in this population, and into how to design a peer support program that truly supports behavior change.

Using SDT as a framework, focus groups have been utilized to study health behaviors in low-income populations including low-income pregnant women who are overweight or obese [18], older adults of differing economic status with barriers to increasing physical activity [19], and low-income Latinx adults with Type II Diabetes [20]. This research consistently finds that SDT is useful for evaluating data on underserved populations. These studies address health-related cardiovascular risks and behaviors such as physical activity and diet similar to those included in our study.

Though focus groups have studied health behaviors in low-income populations, to our knowledge there are no recent studies involving focus groups made up of low-income urban residents, with content that includes all three major determinants of cardiovascular outcomes (nutrition, physical activity, and stress), and the unique challenges that urban populations face. Further, while Self-Determination Theory has been used as a conceptual framework for some focus group studies, it has not been used for focus groups involving our specific population and types of cardiovascular risk. Our study contributes to the existing body of knowledge by using focus groups to explore perceptions of a range of cardiovascular risk-related barriers, strengths, supports, and preferences among a low-income urban population, with SDT as an organizing framework for the findings.

## Methods

### Recruitment and data collection

This is a mixed methods study using primarily qualitative data, with some quantitative data included. The study was approved by the Stockton University and Geisinger/Atlanticare Institutional Review Boards (IRB) and took place in Atlantic City, New Jersey. This northeastern U.S. city has a tourism and casino-based economy which was economically devastated by the closing of multiple casinos in the last two decades [21]. This downturn is reflected in a 2019 individual poverty rate of 37.1%. The overwhelming majority of residents lack a college degree (83.8%); 16.6% lack health insurance; 29.6% identify as white/non LatinX; 35% identify as Black or African American/non LatinX; and 31.1% identify as Latinx [22].

Study participants were recruited at the Atlanticare HealthPlex, a community-based safety net health facility, which includes a Federally Qualified Health Center and a smaller community family medicine center providing charity care. In 2020, 96.2% of patients were at or below 200% of the federal poverty line, including 86.6% of total patients living in poverty.

We used a convenience sample recruited using flyers and posters left in common areas and individual clinics and referrals by medical staff at the facility. Recruitment criteria required participants to be aged 18–85, current patients at the study site, and have one or more self-reported cardiac risk factors (diabetes mellitus, hypertension, coronary artery disease, obesity/overweight).

Three focus groups were conducted in Spring 2018 at the HealthPlex. Focus group sizes were eight, 12, and 13, for a total of 33 participants. This number of participants in each group is within the typical range for focus groups [23]. The number of focus groups was based on available staffing and funds; however the use of two to four groups is fairly common in health research [24–28]. Each group lasted 60–90 min, depending on the length of participant comments. Quantitative data was collected by written survey on-site prior to each focus group, followed by semi-structured focus group questions. Participants received a $25 supermarket gift card as an incentive for taking part in the study.

The written survey, developed for this study by a multidisciplinary program planning group, collected several types of information. The first set of questions focused on demographics and personal characteristics such as self-ratings of finances and established cardiac risk factors. The second set of questions asked about barriers to health behaviors. The third set included questions about program preferences and resources that could impact programming, such as access to smartphones and cooking facilities. The survey items are presented in Additional file 1. Some of the program planning and barriers questions (questions 12, 14, and 25) were adapted from an Atlantic City needs assessment survey previously developed by the Atlanticare Foundation, a charity whose mission includes supporting the wellness of the Atlantic County community. Question 13 was designed by one of the authors (Miserandino) to elicit SDT needs that might be met by the planned program.

The focus groups were conducted by professional facilitators employed by the health care system. Members of the research team were present at each focus group. We used semi-structured interview guides including questions on health care goals, supports, barriers, and suggestions for our future program. Focus groups were digitally recorded and then professionally transcribed. The focus group questions, developed for this study by the multidisciplinary planning group referenced earlier, are presented in Additional file 2.

### Analysis

Quantitative survey data was analyzed using SAS 9.4 software, focusing on descriptive statistics. Qualitative data was analyzed using NVivo 12 qualitative analysis software. Analysis was conducted in two stages. For the first stage, we developed themes without consideration of SDT concepts. In this stage, we used open coding for the first focus group and then discussed and developed a code book to be used for the second and third focus groups. Additional codes were added to the codebook if new topics or themes were identified in the later groups. Paired coding was used for each focus group transcript to ensure rigor. Codes were then discussed and adjusted until agreement of over 95% was reached for each code. After coding comparison, codes were discussed and combined into themes. The second stage of qualitative analysis aligned the themes with SDT concepts, which became the metathemes for the analysis. This two-stage approach allowed us to first capture a broad range of meanings within focus group data, and then use the identified themes to explore how SDT constructs operated within our low-income urban population.

## Results

### Quantitative results

As described earlier, a written survey was administered immediately prior to each focus group. Surveys included questions on demographics, program planning, and barriers and resources.

#### Demographics

Sample demographics and characteristics are presented in Table 1. The study sample size was 33 across the three focus groups. Participants were 63.64% female with the remainder male. An “other” category was offered but no participants selected it. Mean age was 50.80, with a range of 24 to 71. During the focus groups it became clear that a small number of participants came in pairs as either couples, friends, or relatives, however we did not ask about this in the surveys so cannot state the frequency. The largest racial group was African American, with 48.48% of participants identifying in this category. The next largest group was white (27.27%), followed by Latinx (12.12%), other (9.09%), and then Asian (3.03%). Participants were asked to identify as many categories as applied to them, but none chose more than one race/ethnicity. The majority of participants (60.61%) reported incomes of less than $1000 per month. Almost 20% of participants did not select an income level, so it is difficult to ascertain the true percentages for this question.

**Table 1:** Participant characteristics

	Number	Percentage	Mean (SD)
Age	30		50.80 (14.10)
Gender			
Male	12	36.36	
Female	21	63.64	
Race/ethnicity^a^			
Black	16	48.48	
White	9	27.27	
Latinx	4	12.12	
Asian	1	3.03	
Other	3	9.09	
Income source^a^			
Employment/job	4	12.12	
SSI/SSDI	23	69.70	
TANF	0	0.00	
GA	3	9.09	
Family	4	12.12	
SNAP			
Yes	24	75.00	
No	8	25.00	
Health insurance			
Yes	32	96.97	
No	1	3.03	
Monthly income			
0 - $200.00	6	18.18	
$201.00 - $400.00	2	6.06	
$401.00 - $600.00	3	9.09	
$601.00 - $800.00	9	27.27	
$801–$1000	0	0.00	
More than $1000.00	7	21.21	
Not sure/declined/missing	6	18.18	
Overall health			
Poor	8	24.24	
Fair	14	42.42	
Good	8	24.24	
Very Good	2	6.06	
Excellent	1	3.03	
Health problems			
Diabetes	9	27.27	
Hypertension	26	78.79	
Heart disease	4	12.12	
Obesity	15	45.45	
High cholesterol	12	36.36	
Other	5	15.15	
Number of health problems			
1	13	39.39	
2	10	30.30	
3 or More	10	30.30	

^a^Participants could select more than one option

About 12 % (12.12%) of respondents were employed, and 69.70% received some form of disability benefits, specified as Supplemental Security Income (SSI) or Social Security Disability Insurance (SSDI). None received Temporary Assistance for Needy Families (TANF), but 9.09% received General Assistance (GA) for single adults (New Jersey is one of the few remaining states to offer this very limited public benefit for adults without children). About 12 % (12.12%) of respondents received some amount of financial support from family or friends. Three quarters received Supplemental Nutrition Assistance Program (SNAP) benefits. All but one of the participants had health insurance.

The majority of respondents rated their health as Poor (24.24%) or Fair (42.42%). The remainder rated their health as Good (24.24%), Very Good (6.06%), or Excellent (3.03%). The most frequent health conditions identified by the participants included hypertension (78.79%), obesity (45.45%), high cholesterol (36.36%), and diabetes mellitus (27.27%). The largest group of respondents (39.39%) identified only one health problem, followed by 30.30% identifying two health problems, and 30.30% identifying three or more.

#### Program planning survey questions

Participants were asked about what health interventions they would like to see in their communities. More than half endorsed options including exercise activities (66.67%), cooking classes or healthy prepared meals (66.67%), and community gardens or farmers markets (51.52%). Regarding specific program activities they would be interested in, more than half endorsed healthy meals or food baskets (84.85%), learning to relax in stressful situations (60.61%), and cooking demonstrations (54.55%). When asked what program features would be most important in supporting their personal health goals, participants identified “having someone who knows what I’m going through” (57.58%), “people to share the experience with” (54.55%), “a support network so I don’t feel isolated/alone” (51.52%), and “people who won’t judge me” (51.52%). Table 2 presents the full list of program preferences.

**Table 2:** Program planning

	Frequency	Percentage
Health interventions you would like in your community:		
Walking programs/exercise activities	22	66.67
Cooking classes/healthy prepared meals	22	66.67
Community gardens/farmer’s markets	17	51.52
Incentives/coupons	16	48.48
Other	4	12.12
Most important in a program, to meet health goals:		
Someone who knows what I’m going through	19	57.58
People to share the experience with	18	54.55
Support network so I don’t feel isolated/alone	17	51.52
People who won’t judge me	17	51.52
Place where I can be myself and “feel normal”	14	42.42
Place to exercise	14	42.42
Quiet space to relax	12	36.36
Role models I can look up to	8	24.24
As part of this program, I would be interested in:		
Getting healthy meals/baskets of nutritious foods	28	84.85
Learning ways to relax in stressful situations	20	60.61
Cooking demonstrations	18	54.55
Exercise instruction/group class	16	48.48
Yoga/gentle yoga class	14	42.42
Answers to my questions about a healthy diet	14	42.42
Information on ways to exercise on a budget	13	39.39
Tours of local food stores with advice on healthy diet	12	36.36
Not sure	3	9.09

#### Resources and barriers survey questions

Participants often lacked basic resources that are typically needed as part of cardiac prevention programs. Table 3 presents the results for participant resources and barriers. Twenty-one percent lacked an oven and the same percentage lacked a stove for cooking. A third did not have a nearby location where they could buy healthy food. In spite of lacking resources, the majority of respondents (66.67%) stated that they prepared at least one meal a day at home. Just 57.58% of respondents had smartphones. Twelve percent did not have a phone of any type. The majority of respondents stated that they have a safe place to walk or exercise (81.82%), but 9.09% said they did not, and 9.09% were unsure or declined to answer. The biggest barriers to healthy choices cited by participants were transportation (51.52%), cost (48.48%), stress (48.48%), access to healthy food (42.42%), and access to exercise equipment or a place to exercise (33.33%). Responses to questions on health care providers (items 21–25) are not presented. Answers to these questions were inconsistent and it appears that all participants may not have understood them in the same way.

**Table 3:** Resources and barriers

	Number	Percentage
Appliances owned		
Microwave	30	90.91
Stove	26	78.79
Oven	26	78.79
Hot plate	8	24.24
None	1	3.03
Have a phone, any type	29	87.88
Have a smartphone	19	57.58
Safe place to exercise	27	81.82
Place to buy healthy food	20	60.61
Prep one meal daily	22	66.67
Barriers to making healthy lifestyle choices		
Getting there/transportation	17	51.52
Cost of maintaining a healthy lifestyle	16	48.48
Too much stress about housing/family/other problems	16	48.48
Access to healthy food	14	42.42
Access to equipment or place to exercise	11	33.33
Safety or security concerns	6	18.18
Other barrier	3	9.09
Not enough time/too busy	2	6.06

### Qualitative results

We present our qualitative results organized by related SDT constructs, with brief comments linking each theme to its related construct. We will explore these connections more fully in the discussion section.

#### Autonomy

We asked participants about their health-related goals, and occasionally goals were shared as part of responses to other questions. In the discussion section, we will explore how goals can either support or undermine autonomy depending whether they are self-selected (autonomy-supporting) or identified by others (controlled). Participants readily shared goals for positive change. The most prevalent themes were diet and weight loss. Among those who wanted to change their diet, many stated that they wanted to eat more fruits and vegetables. Others described foods they needed to eliminate from their diets. One participant illustrated both of these themes:

“I need to stop eating such fried foods, everything is like fried. I need to start eating more fruits and vegetables. Like I love spinach and eggs, I can eat that almost every other day, I love ‘em. I need to stop frying and stuff all the time with the oil and it’s greasy, it's no good.”

Others wanted to cook more, or gain knowledge about how to prepare and enjoy healthy foods.

Autonomy was also evident in participants’ range of suggestions for activities they would like in a group intervention. Participants were eager to identify the means by which they would like to attain their goals as part of the planned program. For physical activity, yoga was the most common suggestion. Others asked for swimming, tai chi, and group walks. For diet, the most common responses centered on healthy cooking class, nutrition information, recipes, and potluck meals. There were many suggestions for stress reduction, including meditation, music, reading, pet therapy, and art therapy. Some specifically mentioned peer support: *“I think support groups like talking like he said, just support.”*

#### Competence

##### Barriers towards meeting health-related goals

External barriers can undermine competence, because individuals are unable to complete identified tasks. We asked specifically about barriers that impacted diet and physical activity. For diet, by far the most frequently mentioned barrier was the cost of healthy food. The next most common factor was distance to vendors that sold healthy food. These issues frequently overlapped, with the nearby options having either no healthy food, or healthy food that was too expensive for the participants to purchase. As one focus group member stated:

“If there was a market that just had fruits and vegetables that was reasonable that would be good. But if I could say its variety is not there and the cost is too high and it's like I go to [name of supermarket] and get depressed. Oh God it’s like same thing, it’s like I want something different, you know what I mean, and it's not there.”

The next most frequent barrier to eating healthy foods raised by participants was preference, habit, or history. Participants said they just did not like the taste of foods that were recommended to them. They often looked at it as a chore to learn to like these foods: *“I don’t like salad but I know I have to learn how to-- I have to learn how to eat [it].”* Others described growing up eating unhealthy foods.

While family was often a support, several participants noted family responsibilities as making it more difficult to stick with a healthy diet. For example, one stated: *“It’s just hard-it’s hard sticking to that diet. You know, you can get on a roll but you break, holidays come and, you know, the kids come and you gotta...I gotta cook the fries...”*

Only a few participants mentioned lack of knowledge as a barrier to eating better, although several said they would welcome cooking and nutrition information as part of a potential group activity. Medical providers were viewed as a source of knowledge, but were not described as taking participants’ preferences into account. While the provision of knowledge can support competence, the lack of respect for preferences undermines autonomy (as part of goal selection) and relatedness (to medical providers).

The most frequent comments about physical activity barriers centered on participants’ health limitations. Participants described a number of health conditions that typically impact physical activity, including orthopedic injuries, chronic pain, respiratory problems, and obesity. One participant shared:

“I cannot twist, I cannot bend, I cannot stoop down. So, but I ordered a tai chi complete program and whatever I can do because, you know, tai chi is very slooow…I’m excited waiting for it because I used to do yoga but now I'm going for the tai chi. And I would invite any one of you who wants to come and join me, you can come.”

After health limitations, the most commonly shared barriers were similar to those for improving diet: cost and access. Places to exercise were either too expensive or too far away. Transportation overlapped with access, because participants either did not have cars or could not afford transit fare.

Weather also presented a barrier for those in this group who had no place to exercise indoors. One participant whose main exercise was walking outdoors stated: *“You can’t get by, you know. Especially when you have a walker, you know, like, and then, all the snow is so piled up all to-- all over. I mean there’s like… Up, you know. You can’t, there’s no way.”* For these participants, cost and access to indoor exercise locations combined with lack of other resources to make exercise much more difficult.

Several participants mentioned depression as a barrier, and one participant stated that stress lies beneath all of the barriers they experienced: *“See, the real heart of the matter concerning these topics likely, number one, can be summed up, just under stress.”*

##### Successful change and coping strategies

Some participants shared areas of successful change - reflecting competence - for example losing weight, increasing physical activity, or incorporating nutritious foods into their diets. Participants were resourceful in identifying many individual coping strategies, in particular relating to reducing stress. These included activities such as venting, reading, doing puzzles, watching sports, and going outdoors. Some participants stated that caring for others, or even a pet, could be a source of strength for them. Participants identified a number of sources of support, especially friends and family. For example:

“Yes, I go to my sister because I do get stressed and she's the calmer one, she calms me down. [laughter] She's like, ‘It's not that bad. It’s not that--.’ She's like, ‘Breathe, take it easy.’ I'm stressed out and I call her and she gets me back down a level.”

A number of participants found faith as a strong source of support in their lives. This took a range of forms, including praying, reading the Bible, and attending a house of worship. One participant stated: *“So I go to the Bible a lot and I just read it and it calms me down with the things that I'm going through. I do, I just pray.”*

#### Relatedness

##### Connections with others

SDT holds that relatedness can powerfully enhance behavior change, and participants frequently brought up connections with others during the focus groups. These others included family, friends, and medical professionals. Behavior change was sometimes explicitly linked to relationships. For example, one participant spoke of learning how to cook in a more healthy way from a niece:

“Most Black people don’t like to hear cooking collard greens and string beans and stuff without meat, but uh, my niece has taught me how to cook that and they are very tasty and now, I don’t want to cook ‘em with meat it’s uh, and it’s very nutritional and I have come from uh, 232 pounds down to 212.”

Faith was both a coping skill and a source of relatedness. When asked about who they talk to about their health goals, one participant said:” Well, I have church family. I’m involved in my church.” Another spoke of God as someone they could rely on: “I lean-- I just lean on God.”

Isolation can be viewed as the lack of relatedness. A number of participants spoke of the lack of others in their lives, for example: “What I said sometimes when you don’t have nobody and you feel a lot lonely.” Another participant shared: “Surveys have said more seniors die because of loneliness. I always wanted to do like a little party, get together. We dance, we sing, we -- But I don’t have anybody I’m new and I’m not from Atlantic City. I don’t have anybody here…”.

##### Relatedness within the focus groups

During the focus groups themselves, connections appeared to be forming between group members. For example, they frequently asked for and gave each other advice. In addition, there were commonalities that emerged from the group. Participants responded to each other with supportive comments that recognized their shared experiences. Examples include the struggles to maintain a good diet: “You don’t eat a lot of sweets and stuff? Because I’m a diabetic myself so I know how it is.” and managing extended family living together: “Sounds like my house. Got those grown folks in my house, children in my house, I got other people’s children.” Participants spontaneously connected with each other. One participant illustrated this idea by saying “I think we all going through the same thing, sounds like me.”

##### Suggestions for group intervention

Almost all the participants endorsed the idea of a group intervention. They felt that peer support was important, and would help them reach the goals they had shared earlier in the focus group:

“Yeah well, if you want to join together as a goal to lose weight and support each other in the goals as part of maybe the exercise part, weigh-ins and the recipe of the week or whatever's a reason to come together, because it's so much easier to do with someone than it is to do, particularly if you live alone, than you do by yourself, you know.”

Some participants asked if they could sign up for the program on the spot. In their comments about a future intervention, participants connected their focus group experience to anticipation of a future program: *“Having groups like this, this is wonderful.”*

#### Self-determination constructs summary

Table 4 presents participant quotes that illustrate the SDT core constructs of autonomy, competence, and relatedness. The ability to choose from a range of options that are personally appealing and attainable supports autonomy. Several quotes suggest that when participants described strategies they had developed on their own, that aligned with their own needs and preferences, their statements were positive and change-oriented. In contrast, their quotes about providers who counselled them on actions they “have to” take indicated challenges to adherence. Although Table 4 groups statements according to individual SDT constructs, considerable overlap exists. For example, while the lack of variety in available foods undermines the autonomy to make individual choices, the lack of affordability undermines the competence to buy healthy food at all. Additionally, participants described interactions with providers that thematically connected to not feeling listened to and recognized as individuals, which can undermine all three needs.

**Table 4:** Self-Determination Theory constructs

Construct	Undermines this construct (quotes)	Supports this construct (quotes)
Autonomy Definition: The need to choose and pursue goals that are personally meaningful, rather than being given directive advice	“The doctor said, ‘Well you **have to** eat lettuce, tomato,’ whatever they got they call this stuff eating. But I want to be able to do that. The doctor say, **‘Do that.’** I want to do it. But then if I start, I start doing it, lettuce, tomato, all this type of stuff and eating this here. I’m not satisfied.” “Then I got to understand that the doctor tells me, ‘**You have to** slow down on that greasy food.’ I’ve been doing this all my-- eating greasy food all my life.” “I’m **not supposed to have** it, see. But I grew up on salt. Like, I like bacon and stuff like that. I try to calm down with it but I’m going in the refrigerator and getting it.”	“I’ve learnt to-- I’m learning that no, I love rice but then I prepare shredded vegetables with a lick of rice just to, ya know, trick myself. So I have a lick of rice but it’s more vegetables. It’s nice. I’ve done that 3 days now. Three days I have been doing that and I’m going to continue, and I do my own recipes, just invent.” “But also, made a deal with myself actually to cook for myself, because I live alone, and I really, even if I only take 1 day a week to cook some meals.”
	“..they have a farmer market here but I went there last summer and that, the prices was sky high. I went over to [name of supermarket], there’s was a little better but it still wasn’t enough variety in there for **what I was looking for**. So then that makes me go back to the sweets getting the cookies and the candies.”	
Competence Definition: The ability to complete tasks successfully	“I know good food with the right way to eat, it’s just a question of being too-- I don’t know, um, depressed or do whatever to get started with it all.” “I’m suffering from pretty severe chronic depression even with medication, so it’s really getting motivated to get up and get out.”	“I can block out the negative. I’ve learned how to do that.”
	“When I get next to greens and plants and vegetables, I start feeling a little bit concerned and overwhelmed. I haven’t really figured out why.”	“I once thought that I couldn’t really eat without some meat on my plate. But now, I find that I don’t even want meat.”
Relatedness Definition: Feeling connected to others; a sense of belonging	“I know what good eating is, but I’ll tell you what, since I’ve been living alone, I would, if I don’t cook for friends and give the food away and keep some for myself, I don’t cook healthy.”	“Because it will motivate me more if I have somebody that’s, one or two persons, so you can, if you’re interested you can take my number, call me any day. Yes.” “She walks the boards, so she grab me and say, ‘Let’s walk.’”
	“Living alone sometimes is stressful.”	“... cause she likes to just stay home all day and stay in bed all day and watch - she’s just out of everything, but she has started because I told her, ya know, like, ‘Start getting up, just walking around the block, talk to your next-door neighbor, go to—’ So now, she likes to go to Walmart and watch the people she says.”
	“Like they might give [name] one drug, give to her this drug and I say ‘I can’t take that’ but that’s what they order for everybody but I’m not everybody. My body don’t react well to this. Or they say ‘Your blood pressure should norm should be 102 over 56... I said it might be good according to the chart but it doesn’t make me feel good.” “Because anything and everything that you eat you got to literally monitor and when you tell your doctor, they don’t want to hear nothing about it. Now a person like me had to come here and tell ya’ll about that.”	“A doctor that actually cares.” “Listen and learn, listen and learn.”

Note. Bolding added by authors for emphasis

## Discussion

In this study, we aimed to learn the perceptions of health-related needs among low-income urban residents at risk of cardiac disease. We asked about goals, barriers, and sources of strength relating to cardiovascular risk. We additionally sought participant input as part of program planning for a group intervention. We used SDT as the analytic framework for our findings.

### Autonomy and health-related goals

Goals can support or undermine autonomy depending on who generates them. Participants’ primary health-related goals were overwhelmingly related to diet and nutrition. They were aware of the importance of these factors in addressing a significant burden of self-identified fair to poor health. Despite the many day-to-day housing and food insecurity challenges known to exist at this income level, participants maintained a vision of better health and health habits. Their goals generally aligned with well-accepted approaches to reducing cardiovascular risk. In some cases, participants were clearly sharing goals that were self-generated, for example the individual who was “excited” to start tai chi. Other times, participants used wording such as “should” or “need to” reflecting goals that may have been internalized from providers or others and are not necessarily autonomy supportive. When given the opportunity to suggest their own preferences for the means to meet their goals - supporting autonomy - participants were eager to do so. We argue that seeking out and incorporating participant preferences should be an essential component of an autonomy supportive program, and will enhance goal completion. The goals that clients shared as clearly coming from medical providers often did not seem autonomy-supportive. Taste, preference, and habit or history were important to participants in selecting healthy foods and increasing physical activity, yet participant input indicated that their preferences were not being considered in goal setting. Participants faced challenges relating to their need for autonomy, the need to have input into goals, and activities that are personally meaningful. While participants’ goals were consistent with standard recommendations, they needed (due to barriers) and wanted (due to preferences) to design individual strategies to meet them. Advice from healthcare providers seemed to be directive rather than collaborative. This finding aligns with research demonstrating that those in poverty more commonly experience directive medical communications than those of higher socioeconomic status and dominant groups [29]. To support autonomy, and enhance goal attainment, low-income individuals at cardiovascular risk should have a collaborative partnership with their healthcare provider to develop individualized goals that are personally meaningful and realistic for them.

### Competence and barriers

Many of the barriers to healthy diet and exercise identified by participants were directly related to cost. For many, healthy food was nearby but not affordable. This dilemma is captured in Sullivan’s concept of food deserts (lack of supermarkets) versus food mirages (nearby, but unaffordable, markets) [8]. For this group of low-income individuals, problems relating to poverty provided the most powerful barriers to meeting their health goals. Further, participants recognized that stress -- often correlated with poverty -- was a significant barrier to cardiovascular health.

The lack of resources described by our participants aligns with the SDT construct of competence, because the lack of money and accessible resources translates to a lack of tools to complete a desired task. For persons living in poverty, the SDT basic psychological need for competence is undermined because participants do not have the opportunity and resources to attain their goals. Ingrained societal messages frame the inability to attain these goals as personal failures [30], and our participants made statements that suggested they had internalized these assumptions when they indicated that they wanted to follow certain directives that they were unable to. Therefore, the concrete barriers are compounded by the thwarting of the basic psychological needs, most notably competence, that make it difficult to take effective action.

Stress is a key barrier for those in poverty. Illustrative of this is one participants’ statement that stress lies beneath all of the barriers they experienced. Stressors multiply under the constraints of poverty; they are caused by and cause poverty in an iterative fashion. The psychological need of competence is supported by setting attainable goals [31]. Expecting major changes in health behavior without recognizing the challenges caused by stress undermines competence. Providing stress-reducing measures supports this need. Participants clearly believed peer support would empower them to address stress and move forward with positive change.

### Relatedness

Social capital and social networks can be limited in low-income neighborhoods as compared to other neighborhoods [32]. Participant quotes highlight the impact of loneliness, often connected to physical limitations or depression, and shared the importance of relationships in meeting their health-related goals. The focus groups themselves began to turn into de facto support groups, with participants asking for and giving each other advice, and providing each other with emotional support. Participants enthusiastically endorsed the idea of a peer group intervention.

Beyond the peer support built into a group intervention, relatedness needs to be taken into account in group content. For example, our participants shared that families could be instrumental in meeting health goals, including providing recipes and encouraging physical activity. However, they also sometimes presented challenges to making positive changes, as in when children have strong preferences for unhealthy foods. In some cases, advice from peers or family appeared to generate enthusiasm in a way that advice from medical practitioners did not.

Participant quotes about providers expressed perceptions of not being seen as individuals and of not being listened to, and underscored the importance of being cared for by their providers. The lack of individualized care undermines relatedness, and therefore undermines the motivation for making and sustaining the health-related behavioral changes that these participants know how to make and want to make. Both individual health care providers and group facilitators as well need to form supportive relationships with their clients or patients, and consider and support outside relationships when designing interventions, goals, and treatments.

### Implications for program planning

The use of focus groups allows effective identification of the concrete barriers impacting specific populations. Additionally, it supports SDT needs of autonomy, competence, and relatedness by valuing participant input and respecting their choices, thus empowering participants to overcome barriers with creative solutions as a team. Participants suggested activities that would be attainable with their physical limitations, habits, and cultural preferences. Participants often understand these better than practitioners do and can be valuable resources and supports for each other. They embraced group activities that align with research-based approaches e.g. yoga for stress reduction [33], peer support [34], and information-sharing about better nutrition and exercise [35]. Participants’ comments demonstrated a richness of suggestions and an enthusiasm about coming together and supporting each other to explore those suggestions.

The activities listed above were prioritized when we designed our peer support intervention, and other lower-rated activities such as shopping tours were not. In addition, recognizing that autonomy was important to participants, we continued to build flexibility into our curriculum which spoke to the preferences of each individual group. We trained a portion of our team in motivational interviewing techniques including celebrating small gains to support competence. We tailored our program to ensure that needed resources would be available to meet the goals participants identified. We measured perceived competence using established SDT scales as part of pre- and posttests to ensure we were supporting this need.

### Limitations and future research

Our study provides much information about the needs and wishes of low-income urban residents at risk of heart disease. In some cases, our qualitative data suggested additional questions that would have clarified the quantitative survey. The qualitative questions elicited a much more substantial constellation of interrelated barriers than our quantitative data, which targeted a limited number of specific barriers. Follow-up quantitative research could incorporate the qualitative findings to enhance survey research. It is possible that the order of our data collection - individual surveys prior to focus group questions - may have impacted the input given during the focus groups. We believe that obtaining individual responses prior to group responses allowed us to obtain each participants’ preliminary perspectives prior to the group discussions and possible influence. However, conducting a focus group without first implementing the survey would help evaluate whether the survey steered participants toward specific responses.

Our analysis found that SDT constructs are useful in framing the perspectives of participants, however we did not include focus group questions that explicitly asked about SDT core components. Future research that incorporates questions more directly focused on SDT would add to knowledge of how these ideas can be incorporated in program planning. Ideas raised by our participants could provide direction for future research on SDT. For example, using statements by focus group participants to train medical residents in SDT constructs could potentially equip residents in better supporting autonomy, competence, and relatedness in patients. An evaluation of such a program could be a fruitful application of the ideas raised by our participants. Our focus group data was used in designing a cardiac prevention program which is in progress. Results from this research, which specifically asks participants about perceived competence pre and post intervention, will shed light on the effectiveness of a program design based on SDT.

## Conclusions

This focus group research underscores the need for assessing concrete barriers, and SDT-related impacts of our communities’ lived experiences. This is key to planning programs and policies that effectively combat cardiovascular health disparities. Multiple concrete barriers prevent low-income populations from addressing CVD risk. Our focus groups demonstrated how these barriers impacted our study population. At the same time, our participants’ responses indicated a lack of support of the three basic SDT needs of autonomy, competence, and relatedness. While SDT is frequently studied among non-poor populations, we argue that this framework may be even more important in supporting core psychological needs in a low-income population. Because the needs of autonomy, competence, and relatedness are supported or thwarted by the environment or context, poverty and SDT-related barriers are intertwined in a way that is difficult to separate. Poverty-related concrete barriers prevent task completion, and these barriers also make it less likely for individuals to be supported in basic psychological needs. These two types of barriers combine to create disparities in cardiovascular wellness, leading to disability that in turn creates more barriers. Prevention programs for low-income populations must address these concrete barriers in order to be successful. Program staff must also be aware of both the challenges and strengths of low-income neighborhoods, in order to sensitively and effectively design and facilitate program components. At the same time, because persons living in poverty have environments that undermine autonomy, competence, and relatedness in medical settings, it is also essential to support these needs with health-promoting programs and policies that are mindful of SDT constructs. Focus groups of low-income individuals are a way to clarify how these two approaches -- concrete barrier removal and SDT basic needs support -- operate in a particular population. We must hear the voices of our low-income communities to understand their needs and goals for preserving cardiovascular health, both to inform our providers and to create effective risk reduction programs. Concrete barrier removal and SDT must be implemented in tandem in order to truly and equitably support attainment of health-related goals in a low-income population.

## Supplementary Information

**Additional file 1.** Participant Survey Questions.

**Additional file 2.** Focus Group Interview Guide.

## Data Availability

The datasets used and/or analyzed during the current study are not publicly available due to the qualitative nature of our project. Personal narrative can be more easily associated with individuals. Data may be made available from the corresponding author on reasonable request, and approval by our institutional review boards.

## Abbreviations

ACEs: Adverse Childhood Experiences
CVD: Cardiovascular disease
GA: General Assistance
IRB: Institutional Review Board
SDT: Self-Determination Theory
SNAP: Supplemental Nutrition Assistance Program
SSDI: Social Security Disability Insurance
SSI: Supplemental Security Income
TANF: Temporary Assistance for Needy Families
