# Supplementary material for: ‘Listen and learn:’ participant input in program planning for a low-income urban population at cardiovascular risk
Source: BMC Public Health. 2021 Mar 15;21:504. doi: 10.1186/s12889-021-10423-6 (PMC7962280; doi:10.1186/s12889-021-10423-6)
Supplement: Supplementary file 1 — Additional file 1. Participant Survey Questions. [file 12889_2021_10423_MOESM1_ESM.docx]

**‘Listen and Learn:’ Participant input in program planning for a low-income urban population at cardiovascular risk**

**Additional file 1:** Participant survey questions

1. What is your gender?

- Male
- Female
- Other:
- Don’t want to answer

1. What is your age?

___ years old

- Don’t want to answer

1. How do you identify yourself? Please check all that apply.

- White
- Hispanic/Latino
- African American
- Asian
- Other:
- Don’t want to answer

1. What is your zip code? ____________________
2. If you don’t know your zip code, what is the town or city where you live?
3. Where do you get your income? Please check all that apply.

- Job
- SSI/SSDI (disability)
- TANF (cash welfare for families)
- General Assistance (cash welfare for singles)
- Friends/family
- Other:
- Don’t want to answer

1. Do you receive food stamps (SNAP)?

- Yes
- No
- Don’t want to answer

1. Do you have health insurance?

- Yes
- No
- Not sure
- Don’t want to answer

1. Approximately what is your monthly income from all sources (before taxes)?

- 0 to $200
- $201-400
- $401-600
- $601-800
- $801-1000
- More than $1000 per month
- Not sure
- Don’t want to answer

1. How would you describe your overall health?

- Excellent
- Very good
- Good
- Fair
- Poor
- Don’t want to answer

1. Have you been told that you have any of these health problems? Please check all that apply.

- Diabetes
- High blood pressure
- Heart disease
- Obesity/overweight
- High cholesterol
- Not sure
- Other:
- Don’t want to answer

1. Choose up to four types of health interventions you would like to see in your community. Please check all that apply

- Walking programs/organized exercise activities
- Community gardens/farm markets
- Cooking Classes/ Healthy Prepared Meals
- Incentives/coupons to encourage healthier food choices
- Other:
- Not sure
- Don’t want to answer

1. If you were to join a group program to help you meet your personal health goals, which of these things would be important to you? Please check all that apply.

- Having somebody who knows what I'm going through
- A support network so that I don’t feel isolated/alone
- People who won't judge me
- People to share the experience with
- Role models I can look up to
- A place where I don't have to pretend, where I can be myself where I “feel normal”
- A quiet space to relax
- A place to exercise

1. As part of this program, I would be more interested in:

- Learning ways of relaxing in stressful situations
- Attending a yoga/gentle yoga class
- Getting exercise instruction/group exercise classes
- Getting information on local places to exercise on a budget
- Attending cooking demonstrations
- Getting a healthy meal/baskets of nutritious foods
- Getting answers to my questions about a healthy diet
- Taking tours of local food stores with advice on healthy choices
- None of these
- Not sure
- Don’t want to answer

1. Which of the following do you have? Please check all that apply.

- Microwave
- Stove
- Hotplate
- Oven
- Don’t have any of the above
- Don’t want to answer

1. Do you prepare at least one meal a day at home?

- Yes
- No
- Don’t want to answer

1. Do you have a place nearby to buy healthy food?

- Yes
- No
- Don’t want to answer

1. Do you have a safe place to walk or exercise?

- Yes
- No
- Not sure
- Don’t want to answer

1. Do you have a phone?

- Yes
- No
- Don’t want to answer

1. If you do, is it a smartphone?

- Yes
- No
- Not sure
- Don’t want to answer

1. In general, do you think your doctor or nurse practitioner listens to you?

- Yes
- No
- Not sure
- Don’t want to answer

1. Did you share your health goals with your doctor or nurse practitioner?

- Yes
- No
- Not sure
- Don’t want to answer

1. If yes - did your doctor or nurse practitioner make a plan that helps you meet the health goals that are important to you?

- Yes
- No
- Not sure
- Don’t want to answer

1. If your doctor or nurse practitioner made a plan, is it hard for you to follow?

- Yes, very hard to follow
- Yes, somewhat hard to follow
- No, I can easily follow
- Not sure
- Don’t want to answer

1. What do you think are the top things getting in your way of making healthy lifestyle choices? Please check all that apply.

- Not enough time/too busy
- Getting there/transportation
- Safety or security concerns
- Limited access to healthy food such as fruits and vegetables
- Cost of maintaining a healthy lifestyle, e.g. food expenses, cost of health clubs
- Limited access to exercise equipment/places to exercise
- Too much stress about financial/housing/family/other problems
- Other:

**Abbreviations:**

**SNAP:** Supplemental Nutrition Assistance Program

**SSDI:** Social Security Disability Insurance

**SSI:** Supplemental Security Income

**TANF:** Temporary Assistance for Needy Families
