# Supplementary material for: ‘Listen and learn:’ participant input in program planning for a low-income urban population at cardiovascular risk
Source: BMC Public Health. 2021 Mar 15;21:504. doi: 10.1186/s12889-021-10423-6 (PMC7962280; doi:10.1186/s12889-021-10423-6)
Supplement: Supplementary file 2 — Additional file 2. Focus Group Interview Guide. [file 12889_2021_10423_MOESM2_ESM.docx]

**‘Listen and Learn:’ Participant input in program planning for a low-income urban population at cardiovascular risk**

**Additional file 2.** Focus group interview guide

**Introduction**

Welcome. You’ve been invited to be part of something we call a focus group. As part of this group, you will answer a number of questions. Your responses to these questions will help us plan a program for HealthPlex patients who have heart disease, diabetes, high blood pressure or are struggling to control their weight. We want to find out what type of services and activities might help patients take care of their health in the way they would like. Your ideas will help us plan a great program and we thank you for joining us today.

You don’t have to participate. Participation is optional, and you do not have to answer every question. All responses given today will be included in a summary report. At no time in this report will we use your individual name or identify your specific comments.

*[Consent and written survey administered here]*

Thank you for completing the survey form. Now we’re ready to get started with the first focus group questions. Here we go.

**Questions**

| Focus Area | Questions |
| --- | --- |
| Health Goals | 1. What are your goals for your health? Why? |
| Social Support/Stress | 2. Besides your doctor or nurse practitioner, is there anyone in your life who you can go to for support with your health goals?  3. Is there anyone in your life that you feel you can talk to when you feel stressed? |
| Nutrition | 4. Is changing your diet important to you? Why or why not?      Probe: What are some of the things that make changing your diet hard? |
| Exercise | 5. Do you think exercise is important for you? Why or why not?      Probe: What makes it hard to exercise? |
| Program Planning    General | 6. As we said earlier, we are planning a group to help people meet their health goals.  If we were to offer such a program, do you think it would be helpful to you? Why?      Probes: How could a group help you meet your goals about eating better?                   About exercise?                   About stress?  7. Are there any specific things about groups/group meetings that you think we should avoid?  8. Is there anything else in general that we haven’t asked, that you think we should know? |

**Conclusion**

We really appreciate your participating. You are helping us develop the new program. If you would like information about the program once it begins, please sign the form and include your contact information. Thanks again!
